# Analysis of Cross-Association between mRNA Expression and RNAi Efficacy for Predictive Target Discovery in Colon Cancers

**DOI:** 10.3390/cancers12113091

**Published:** 2020-10-23

**Authors:** Euna Jeong, Yejin Lee, Youngju Kim, Jieun Lee, Sukjoon Yoon

**Affiliations:** 1 Research Institute of Women’s Health, Sookmyung Women’s University, Seoul 04310, Korea; eajeong@sookmyung.ac.kr; 2 Department of Biological Sciences, Sookmyung Women’s University, Seoul 04310, Korea; cs00800@sookmyung.ac.kr (Y.L.); yjyj0308@sookmyung.ac.kr (Y.K.); rtjklgg05@sookmyung.ac.kr (J.L.)

**Keywords:** gene expression, shRNA screening, CRISPR, anticancer targets, biomarkers, precision medicine

## Abstract

**Simple Summary:**

This study focused on finding genes for which mRNA expression was able to predict the anticancer efficacy of its RNAi treatment. Predictive target discovery is of critical importance in developing biomarker-based strategies of precision medicine. We demonstrated this carrying out cross-association analysis on collateral mRNA expression and RNAi treatment data of ~12,000 genes on a colon cell line panel. The analysis revealed several genes with significant association between mRNA expression level and the inhibitory efficacy of its RNAi treatment. The experimental validation confirm that this simple approach has general applications for studying gene association between omics data from diverse cancer lineages.

**Abstract:**

The availability of large-scale, collateral mRNA expression and RNAi data from diverse cancer cell types provides useful resources for the discovery of anticancer targets for which inhibitory efficacy can be predicted from gene expression. Here, we calculated bidirectional cross-association scores (predictivity and descriptivity) for each of approximately 18,000 genes identified from mRNA and RNAi (i.e., shRNA and sgRNA) data from colon cancer cell lines. The predictivity score measures the difference in RNAi efficacy between cell lines with high vs. low expression of the target gene, while the descriptivity score measures the differential mRNA expression between groups of cell lines exhibiting high vs. low RNAi efficacy. The mRNA expression of 90 and 74 genes showed significant (*p* < 0.01) cross-association scores with the shRNA and sgRNA data, respectively. The genes were found to be from diverse molecular classes and have different functions. Cross-association scores for the mRNA expression of six genes (*CHAF1B, HNF1B, HTATSF1, IRS2, POLR2B* and *SATB2*) with both shRNA and sgRNA efficacy were significant. These genes were interconnected in cancer-related transcriptional networks. Additional experimental validation confirmed that siHNF1B efficacy is correlated with *HNF1B* mRNA expression levels in diverse colon cancer cell lines. Furthermore, *KIF26A* and *ZIC2* gene expression, with which shRNA efficacy displayed significant scores, were found to correlate with the survival rate from colon cancer patient data. This study demonstrates that bidirectional predictivity and descriptivity calculations between mRNA and RNAi data serve as useful resources for the discovery of predictive anticancer targets.

## 1. Introduction

The discovery of biomarkers that can predict the outcome of cancer therapies is important for selecting patients who might benefit from a treatment in current precision medicine strategies [1,2,3]. Although mutations in oncogenes have served as essential biomarkers for targeted drug development and clinical application [4,5,6,7,8], the discovery of additional new mutations as biomarkers is limited due to the low frequency of common types of mutations among cancer samples. More recently, biomarkers obtained from gene expression data, such as PD-L1 and PD-1, were clinically proven to be effective for targeted immune therapies [9,10,11]. Simple gene expression measures have clear advantages over the use of rare or varied mutations in a given gene for association studies of drug efficacy. Thus, transcriptome-oriented screening of biomarkers is becoming a promising approach for finding new gene targets whose expression can predict anticancer efficacy.

The availability of parallel RNA sequencing datasets and RNAi screening data from a large collection of cell lines [12,13,14] provides unique resources for studies on the association between gene expression and knockdown/knockout efficacy in diverse cancer cell types. In this study, we attempted to analyze shRNA and sgRNA screening data from colon cancer cell line panels with RNA sequencing data from the corresponding cell lines. Correlation coefficient measures, such as Pearson’s correlation coefficient and Spearman’s rank correlation coefficient, have been widely used to quantify the association of two variables among samples [15,16,17,18]. However, these measures are inappropriate for quantifying the difference in variables between groups [19,20]. Their ability to describe the association when variables are unidirectionally related between groups is also limited. We thus calculated the bidirectional, cross-association patterns for two variables, mRNA expression and RNAi efficacy data. The predictivity of a gene measures the difference in RNAi efficacy between groups of cell lines with high and low expression of the target gene. In contrast, descriptivity measures the difference in gene expression between cell lines in which the efficacy of RNAi of the gene is high and low. These two cross-association measures indicate the sensitivity and specificity of the predictive power of gene expression versus RNAi efficacy in diverse cancer cell types.

We evaluated the predictive power of gene expression for the knockdown (i.e., shRNA) and knockout (i.e., sgRNA) efficacy for over 12,000 and 18,000 genes in 42 and 35 colon cancer cell lines, respectively. We comparatively analyzed the functional diversity of hit genes for which mRNA expression displayed significant predictive power for knockdown and/or knockout efficacy. The results revealed diverse potential anticancer target genes, the varied knockdown and/or knockout efficacies among cells for which were directly associated with their mRNA expression levels. We believe that this study provides new tools for the simultaneous discovery and evaluation of biomarkers and targets for cancer precision medicine.

## 2. Results

### 2.1. Overall Relationship between RNAi Efficacy and mRNA Expression Level

**Figure 1:**
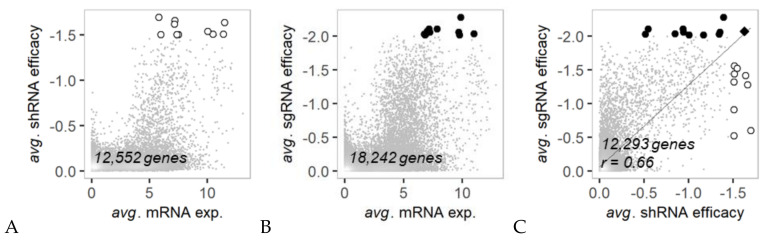
Distribution of genes by average mRNA expression vs. average RNAi efficacy in colon cancer cell lines. (**A**) Average mRNA expression and average shRNA efficacy were calculated and compared using 42 colon cancer cell lines. Empty circles represent the top 10 genes with the highest shRNA knockdown efficacy (<−1.5). (**B**) Average mRNA expression and average sgRNA efficacy were calculated and compared using 35 colon cancer cell lines. Filled circles represent the top 11 genes for which the sgRNA knockout efficacy was highest (<−2). (**C**) Average shRNA efficacy and average sgRNA efficacy were compared for 12,293 genes. A common hit gene from (**A**,**B**) is represented by a rhombus shape. The list of 20 hit genes can be found in Table S1.

Over 12,000 genes were analyzed for their average mRNA expression in comparison with the average antiproliferative RNAi efficacy against the genes in 35 to 42 colon cancer cell lines (Figure 1). Generally, genes with below-average mRNA expression exhibited relatively low anticancer efficacy by shRNA or sgRNA transfection (Figure 1A,B). A total of 10 shRNAs and 11 sgRNAs with a top-ranked anticancer response were found among genes displaying above average mRNA expression. This pattern indicates that a phenotypic impact is typically expected for the knockdown or knockout of genes with high mRNA expression. A list of the top-ranked genes is available in Table S1. Except for one common gene (*SNRPD1*), the top-ranked genes in terms of anticancer efficacy were inconsistent for analyses of shRNA and sgRNA transfection. However, the shRNA and sgRNA efficacies for 12,293 genes showed a cross-correlation (Pearson’s correlation coefficient (PCC); *r* = 0.66) with each other in colon cancer cell lines (Figure 1C). This implies that gene knockdown (shRNA) and knockout (sgRNA) are comparable tools for large-scale screening and/or validation of the anticancer efficacy of target genes.

**Figure 2:**
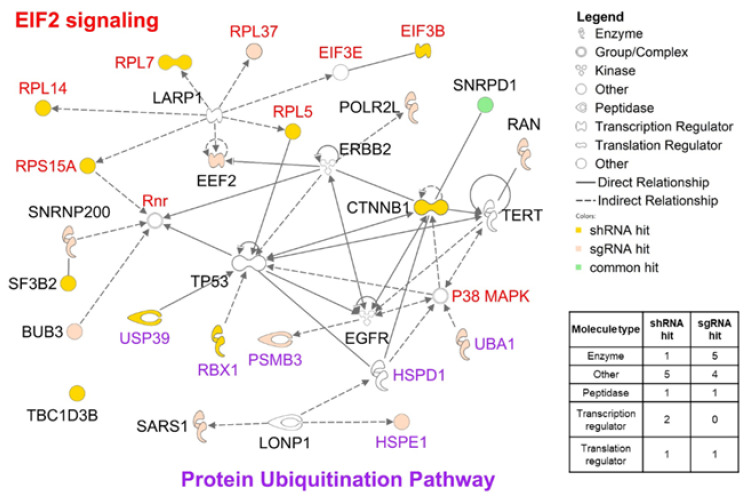
Functional interaction network and molecular classes of the 20 genes with high RNA*i* efficacy from Figure 1. Red and purple gene names indicate genes involved in ‘EIF2 signaling’ and the ‘Protein Ubiquitination Pathway’, respectively. The network and molecular classes were derived using the Ingenuity Pathway Analysis (IPA) tool.

All 20 top-ranked hit genes in terms of shRNA/sgRNA efficacy were found to be involved in essential functions, such as transcription, translation and protein metabolism (ubiquitination) (Table S1). In particular, most of the top-ranked genes in terms of shRNA and sgRNA efficacy are involved in the EIF2 signaling and protein ubiquitination pathways (Figure 2). In terms of molecular function, the top-ranked anticancer genes in terms of shRNA and sgRNA efficacy differed. Half of the genes for which sgRNA efficacy was high (*POLR2L*, *RAN*, *SARS1*, *SNRNP200*, and *UBA1*) were found to be enzymes, while only one gene for which shRNA efficacy was high (*RBX1*) is an enzyme (Figure 2). Most of the top-ranked genes in terms of shRNA efficacy were found to have diverse molecular functions. Functional analysis showed that shRNAs and sgRNAs exclusively display anticancer efficacy against target genes of different molecular types, while functional networks consisting of the target genes overlap in the transcription, translation and ubiquitination pathways.

### 2.2. Predictivity and Descriptivity of Gene Expression for Anticancer RNAi Efficacy

**Figure 3:**
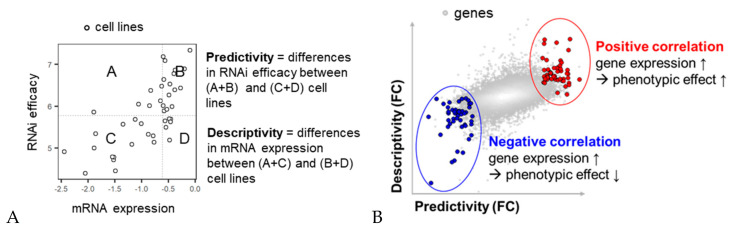
Schematic description of the bidirectional calculation method for the association between mRNA expression and RNAi efficacy for a given gene in the cell line panel. (**A**) Distribution of cell lines by mRNA expression vs. RNA*i* efficacy for a given gene. Predictivity and descriptivity were calculated after cell lines were grouped based on median mRNA expression levels and RNA*i* efficacy. (**B**) An example plot of the predictivity and descriptivity scores of over 12,000 genes derived from the calculation method in (**A**). Colored circles represent genes with statistical significance (*p*-value < 0.01) for both predictivity and descriptivity.

We further analyzed the association between shRNA/sgRNA efficacy and the expression levels of target genes in diverse colon cancer cell lines. The cell lines were classified into four groups based on median mRNA expression levels and the shRNA/sgRNA efficacy for a given target gene (Figure 3A). The predictivity score for a given gene represents the fold change in its shRNA or sgRNA efficacy between cell lines with high and low mRNA expression of the gene (see details in the Materials and Methods section). The descriptivity score for a given gene represents the fold change in its mRNA expression between cell lines in which shRNA/sgRNA efficacy are high and low. These two scores compensate for each other in terms of sensitivity and specificity for the association between mRNA expression level and shRNA/sgRNA efficacy among diverse cell lines. We attempted to calculate bidirectional predictivity and descriptivity scores for each of over 12,000 genes with a statistical test (i.e., *p*-value) (Figure 3B). The resulting 2D plot shown in Figure 3B indicates the comparative consistency between the predictivity and descriptivity of a gene, together with the overall distributions of predictivity and descriptivity for over 12,000 genes.

**Figure 4:**
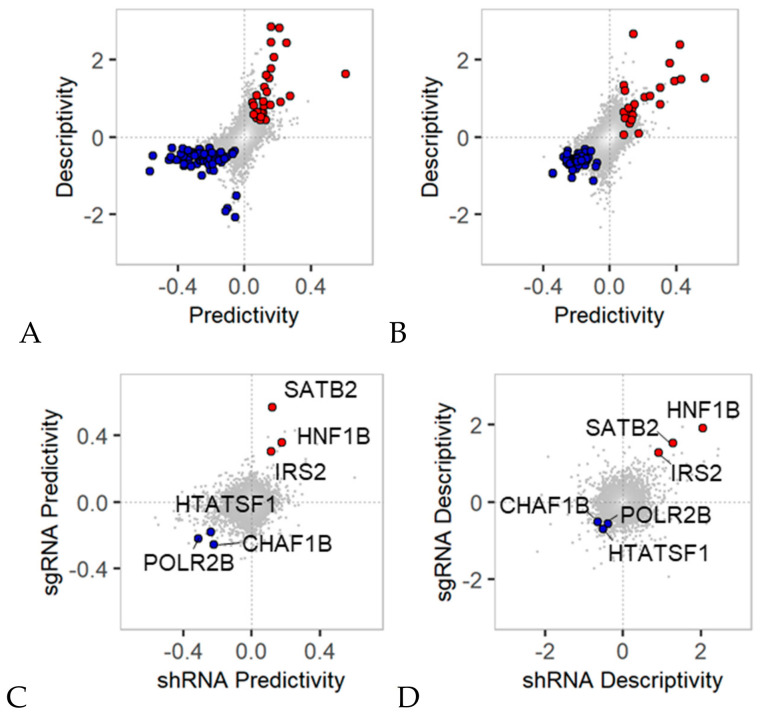
Bidirectional predictivity and descriptivity analyses of gene expression in colon cancer cell lines. (**A**,**B**) Predictivity vs. descriptivity between mRNA expression and shRNA/sgRNA efficacy are plotted. Hit genes showing positive or negative scores with a *p*-value < 0.01 in terms of both predictivity and descriptivity were selected. The numbers of positive hits from the shRNA and sgRNA analyses were 30 and 23 (red circles), respectively, and the numbers of negative hits from the shRNA and sgRNA analyses were 60 and 51 (blue circles), respectively, as shown in the plots. (**C**,**D**) Common hits obtained from shRNA and sgRNA analyses were comparatively plotted in predictivity and descriptivity plots. The common hit genes with positive scores (red circles) are HNF1 homeobox B (*HNF1B*), SATB homeobox 2 (*SATB2*) and insulin receptor substrate 2 (*IRS2*). The common hits genes with negative scores (blue circles) are chromatin assembly factor 1 subunit B (*CHAF1B*), HIV-1 Tat specific factor 1 (*HTATSF1*), and RNA polymerase II subunit B (*POLR2B*).

In the panel of 45 colon cancer cell lines, a total of 90 genes were found to satisfy *p*-value < 0.01 in both predictivity and descriptivity scores between mRNA expression and shRNA efficacy (Figure 4A). Thirty genes (red circles in Figure 4A) displayed positive scores in terms of both predictivity and descriptivity, while 60 genes (blue circles in Figure 4A) showed negative scores. Interestingly, these 90 hit genes showed a nonlinear distribution in a plot of predictivity vs. descriptivity. Hit genes with positive scores showed a higher descriptivity score than predictivity score, while hit genes with negative scores showed the opposite pattern.

This analysis reveals two different aspects of the relationship between mRNA expression and shRNA efficacy for a gene. For hit genes with positive scores in terms of both predictivity and descriptivity, the difference in shRNA efficacy between cell lines with high and low mRNA expression was relatively minimal (i.e., minimal predictivity score), while the difference in mRNA expression between cell lines for which shRNA efficacy for a gene was high and low was relatively large (i.e., relatively high descriptivity score). In contrast, hit genes with negative scores in terms of predictivity and descriptivity mostly exhibited greater predictive power than descriptive ability. This observation implies that hit genes displaying upregulated mRNA expression might be essential for explaining differences in shRNA efficacy among diverse cell lines. On the other hand, downregulated hit genes serve as better biomarkers than upregulated genes for predicting shRNA anticancer efficacy among cell lines.

In an analysis of the sgRNA efficacy for a gene compared with its mRNA expression, the predictivity and descriptivity scores showed a more linear relationship than those obtained by analysis of shRNA efficacy compared with mRNA expression (Figure 4B). A total of 74 genes were found to satisfy the criterion *p*-value < 0.01 in terms of both predictivity and descriptivity scores for mRNA expression and sgRNA efficacy. Hit genes with negative scores (blue circles in Figure 4B) showed minimal predictivity and descriptivity scores. Six common hit genes (*CHAF1B, HNF1B, HTATSF1, IRS2, POLR2B,* and *SATB2*) were identified from analyses of shRNA and sgRNA efficacy (Figure 4C,D). The predictivity and descriptivity of the expression of these genes are significant in terms of both knockdown and knockout efficacy, although the descriptivity scores for negative hit genes (blue circles in Figure 4D) were minimal upon analyses of both shRNA and sgRNA.

### 2.3. Functional Network Analysis of Predictive Target Genes

**Figure 5:**
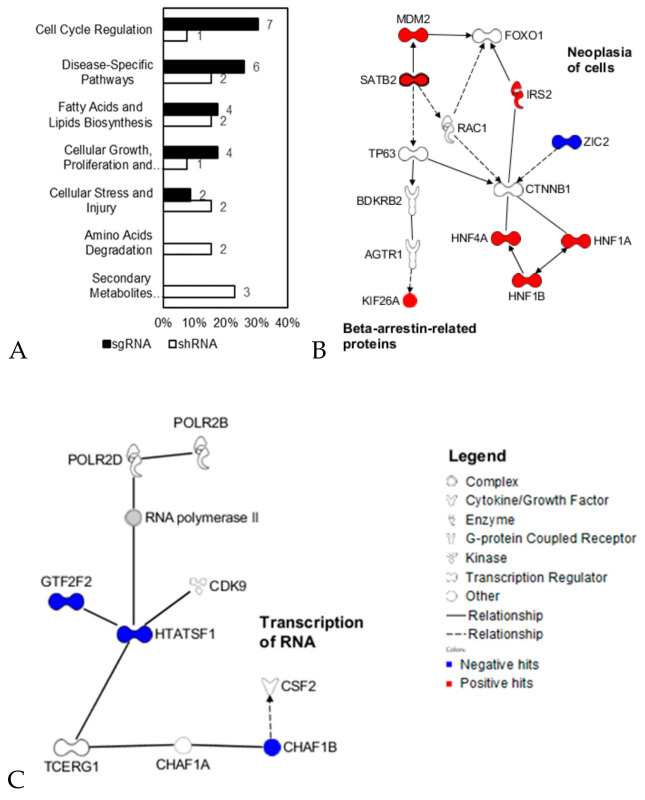
Functional category and network analyses of predictivity and descriptivity hit genes from Figure 4. (**A**) Distribution of functional categories enriched in shRNA and sgRNA hit genes. (**B**,**C**) Network analysis of common hit genes from shRNA and sgRNA analyses. Positive hit genes (*HNF1B,* *IRS2, MDM2* and *SATB2*) are related to ‘neoplasia of cells’, and *AGTR1* and *KIF26A* are related to ‘beta-arrestin-related protein’, while negative hit genes (*CSF2, CDK9, GTF2F2* and *HTATSF1*) are related to ‘transcription of RNA’. The analyses were carried out using IPA software.

In calculations of the predictivity and descriptivity scores shown in Figure 4A,B, the major functional categories related to hit genes obtained from shRNA and sgRNA analyses differed (Figure 5A). shRNA hits were found to be related to only metabolism-related categories, while more sgRNA hits than shRNA hits were related to the cell cycle and disease pathways. This finding indicates that shRNAs and sgRNAs enrich predictive (or descriptive) targets in different functional classes.

We also analyzed the functional networks enriched in common hits identified by analyses of shRNA and sgRNA efficacy (Figure 5B,C). Chromatin assembly factor 1 subunit B (*CHAF1B*) is required for the assembly of histone octamers onto newly replicated DNA. HNF1 homeobox B (*HNF1B*) encodes transcription factor 2. HIV Tat-specific factor 1 (*HTATSF1*) is another transcription factor. Insulin receptor substrate 2 (*IRS2*) is a molecular adaptor between several receptor tyrosine kinases and downstream effectors. DNA-directed RNA polymerase II subunit RPB2 (*POLR2B*) is the polymerase responsible for synthesizing messenger RNA in eukaryotes. Special AT-rich sequence-binding protein 2 (*SATB2*) is involved in transcriptional regulation and chromatin remodeling. Kinesin Family Member 26A (*KIF26A*), as other kinesins, move along microtubule (MT) filaments and supports several cellular functions including mitosis, meiosis. Zinc finger protein ZIC2 (*ZIC2*) has been found to interact with *TCF7L2*, enabling it to act as a Wnt/β-catenin signaling inhibitor, thus regulating Wnt signaling which has been found to be upregulated in several cancers. Positive hit genes were interconnected in the functional networks “Neoplasia of cells” (*HNF1B, IRS2, MDM2* and *SATB2*) and “beta-arrestin-related protein” (*AGTR1* and *KIF26A*), while negative hits were clustered in the functional networks “Transcription of RNA” (*CSF2, CDK9, GTF2F2* and *HTATSF1*). This analysis suggests that the varied expression levels of hit genes in these common functional networks provide statistically significant power for predicting anticancer efficacy by either shRNA or sgRNA in diverse colon cancer cell lines.

**Figure 6:**
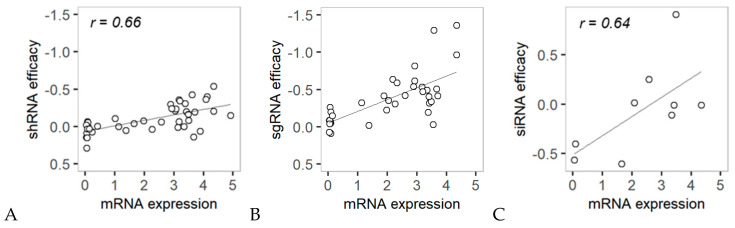
Comparison of *HNF1B* mRNA expression vs. RNAi efficacy in different colon cancer cell lines. (**A**,**B**) *HNF1B* mRNA expression vs. shHNF1B/sgHNF1B efficacy in 42/35 colon cancer cell lines. (**C**) Experimental validation of *HNF1B* mRNA expression and siHNF1B efficacy in nine colon cancer cell lines.

We carried out experimental validation of the association between mRNA expression and anticancer RNAi efficacy with the *HNF1B* gene. As shown by the shRNA and sgRNA analyses, *HNF1B* mRNA expression was significantly correlated with shRNA (PCC; *r* = 0.66) and sgRNA (PCC; *r* = 0.69) efficacy in colon cancer cell lines (Figure 6A,B). We experimentally tested the siRNA efficacy for *HNF1B* in nine colon cancer cell lines and observed that the correlations between mRNA expression and siRNA efficacy for the *HNF1B* gene were similar (Figure 6C). This result confirms the utility of our predictivity and descriptivity calculations for the discovery of anticancer target genes whose mRNA expression is predictive of RNAi efficacy. In the case of the *HNF1B* gene, the predictive power was more obvious with sgRNA efficacy than with shRNA efficacy (Figure 4C), although the correlation coefficients (r) were similar (Figure 6A,B). The difference in sgRNA efficacy (Figure 6B) among the cell lines was greater than the difference in shRNA efficacy (Figure 6A), resulting in a greater predictivity score, as shown in Figure 4C.

### 2.4. Survival Analysis of Predictive Anticancer Target Genes

**Figure 7:**
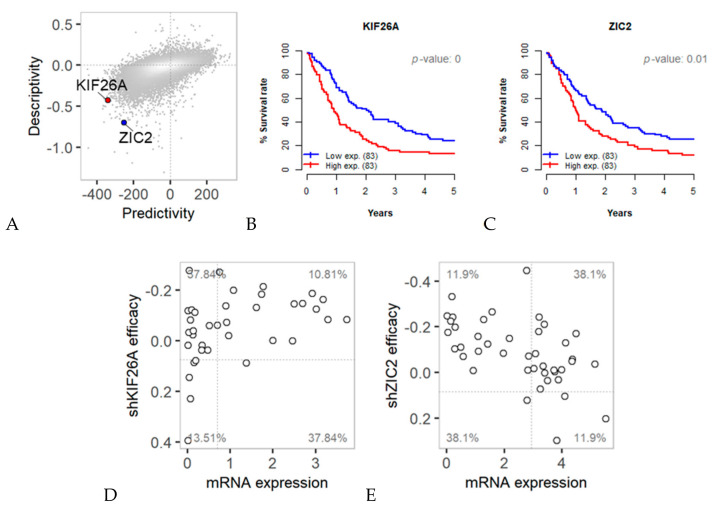
Bidirectional predictivity and descriptivity analyses of 19,079 genes in samples from 171 COAD TCGA patients. mRNA expression of a gene and the survival record of the patient from which the sample was obtained were used for bidirectional calculations. (**A**) Predictivity vs. descriptivity between mRNA expression and disease-free survival in days are plotted. Genes showing positive or negative association with a predictivity *p*-value < 0.01 and descriptivity *p*-value < 0.05 were selected. Among 322 hit genes, *KIF26A* and *ZIC2* are common among shRNA hit genes and showed positive and negative correlations in shRNA analysis, respectively. (**B**,**C**) Graph of disease-free survival according to high and low gene expression of *KIF26A* and *ZIC2*, respectively. (**D**,**E**) mRNA expression vs. shKIF26A/shZIC2 efficacy in colon cancer cell lines.

Furthermore, we calculated predictivity and descriptivity scores for the mRNA expression of the whole transcriptome against patient survival data using TCGA data (Figure 7A). Two shRNA hits (*KIF26A* and *ZIC2*) showed significant (*p*-value < 0.01) scores upon assessment with the survival data. Patients whose samples showed high expression of these genes showed a lower survival rate than those whose samples showed low expression of these genes (Figure 7B,C). However, two genes showed opposite associations between gene expression and shRNA efficacy. *KIF26A* expression was positively correlated with the efficacy of knockdown by shRNA (Figure 7D), while *ZIC2* expression exhibited a negative correlation (Figure 7E). Combined with the survival data, these data allowed us to narrow down the predictive target genes with clinical relevance.

## 3. Discussion

Phenotypic effect of RNAi treatment on cancer cells is caused by inhibiting the mRNA expression level of the target gene. However, no systematic studies have been reported on the relationship between mRNA expression and RNAi efficacies for whole target genes. We have previously showed that of target protein on a large collection of cancer cell lines [7]. It implied that the regulation of mRNA expression of the target genes might partially contribute to the anticancer efficacy on cancer cells. 

In this study, we investigated the association of mRNA expression of approximately 12,000 genes with their RNAi efficacy on cancer cells using a bidirectional cross-association analysis. The results revealed a total of 90 and 74 genes of which the mRNA expression was significantly (*p*-value < 0.01) predictive to their shRNA- and sgRNA-treated efficacies, respectively. Although, genes with low mRNA expression generally exhibited relatively low anticancer efficacy by shRNA or sgRNA transfection (Figure 1A,B), many of genes did not show significant predictive or descriptive association between mRNA expression and RNAi efficacies on diverse colon cancer cell lines. 

We also comparatively analyzed the efficacy data of shRNA and sgRNA with the mRNA expression level. shRNAs and sgRNAs provide different regulation mechanism (i.e., knockdown vs. knockout) on RNA expression, thus typically exhibiting inconsistent phenotypic outcome from each other [21,22,23]. In this study, we found that the mRNA expression of six genes (*CHAF1B, HNF1B, HTATSF1, IRS2, POLR2B* and *SATB2*) showed significant predictivity and descriptivity scores with both shRNA and sgRNA efficacies (Figure 4C,D). Roles of these genes have been widely reported as regulators of cancer progression or drug resistance [24,25,26,27], we suggest here that their mRNA expression level is directly associated with their knockdown and knockout efficacies. Many of shRNA and sgRNA hit genes were found from different functional categories, although they were interconnected in cancer-related signaling networks (Figure 5A,B). This systematic analysis on about 12,000 genes provide a guideline to the utility of gene expression for predicting anticancer efficacy of the target gene.

We used two complementary measures, predictivity and descriptivity scores, to quantify the association between mRNA vs. shRNA data. In case of shRNA data analysis with mRNA expression, significant hit genes from the predictivity measure was quite inconsistent with hits from descriptivity measure (Figure 4A). This discrepancy represented the sensitivity and specificity of the association between mRNA and shRNA data. Considering these bidirectional measures together, we could obtain robust hit genes of which mRNA expression is predictive and descriptive biomarker to the to the RNAi efficacy. 

This bidirectional cross-association measures can be applied to any two collateral omics datasets. We applied this method to the mRNA expression and survival rate data from patient samples (Figure 7). The expression of two hit genes (*KIF26A* and *ZIC2*) from the analysis of mRNA vs. shRNA data, were also found to be significantly associated to the patient survival rate. This result provides new insights on predictive targets with prognostic values in colon cancer therapies. 

Our approach is simple and easy way to study association patterns of a gene between two data sets made from diverse cancer samples. We currently implement this bidirectional calculation method in to a user-friendly tool including diverse cancer omics datasets from multiple lineages. The tool. It will be available from the website (http://qomics.sookmyung.ac.kr) in near future.

## 4. Materials and Methods

### 4.1. Acquisition of Data from Colon Cancer Cell Lines

Large-scale data consisting of shRNA screening data (Combined RNAi), sgRNA screening data (CRISPR, version 20Q1), and RNA sequencing data (Expression, version 20Q1) were obtained from the DepMap portal [28]. For the three datasets (shRNA, sgRNA, and RNAseq), the values were all on a log2-scale, and the data were collected from 44, 42, and 66 colon cancer cell lines, respectively. For this study, the numbers of colon cancer cell lines from which both shRNA and RNAseq data and both sgRNA and RNAseq data were obtained were 42 and 35, respectively. For further analysis, shRNAs and sgRNAs with no efficacy data from more than 50% of the colon cancer cell lines were filtered out.

### 4.2. Acquisition of Data from Patients with Colon Cancer

RNA sequencing data and clinical data were obtained from the TCGA GDC data portal [29]. All of patient sample data in this resource were publicaly available for analyses and further processing. We selected 171 patients with records over five years among 382 patients with colon cancers. The RNA sequencing data in FPKM were converted to log2 (TMP+1) scale. For calculations of relapse-free survival (RFS), we obtained the following patient details: vital status, pathologic stage, days to death, days to last follow-up and days to new tumor event after initial treatment from clinical data. Since we obtained the patient sample data from publicly available open resource, we do not provide any documentation of ethical issues.

### 4.3. Bidirectional Analysis

For bidirectional analysis, we defined two measures: “predictivity” and “descriptivity”. First, predictivity for the cell line data was defined as follows:

Predictivity = avg(phenotypic efficacy in high.exp) − avg(phenotypic efficacy in low.exp)(1)

where phenotypic efficacy was the shRNA knockdown or sgRNA knockout score. High.exp and low.exp represent cell lines with gene expression > and ≤ the median gene expression in all cell lines, respectively. Therefore, predictivity was the fold change between phenotypic efficacy averages (avg) for the two groups based on gene expression, i.e., the high.exp and low.exp groups of cell lines. Similarly, the predictivity of patient data was defined as follows:

Predictivity = avg(alive days in high.exp) − avg(alive days in low.exp)(2)

In TCGA patient data, phenotypic efficacy was the number of alive days without relapse. High.exp and low.exp represent patients with gene expression > and ≤ the median gene expression among all patients, respectively.

Second, descriptivity was defined as follows:

Descriptivity = avg(gene exp. in high.phe) − avg(gene exp. in low.phe)(3)

where gene exp. is the gene expression value, and high.phe and low.phe are cell lines with phenotypic efficacy > and ≤ the median phenotypic efficacy among all cell lines, respectively. Therefore, descriptivity was the fold change in average gene expression for the two groups classified based on phenotypic efficacy, i.e., high.phe and low.phe. Similarly, the descriptivity of patient data was defined as follows:

Descriptivity = avg(gene exp. in long.alive) − avg(gene exp. in less.alive)(4)

where long.alive and less.alive represent patients with a number of alive days > and ≤ the median number of alive days among all patients, respectively.

### 4.4. Biological Functional Analysis and Network Construction

Ingenuity pathway analysis (IPA) was used for network analysis via a license to Ingenuity Systems.

### 4.5. Cell Culture

The HCT 116, HT-29, and KM12 cell lines were purchased directly from the National Institutes of Health, National Cancer Institute (NCI, Frederick, MD, USA); the SW480, SW403, SNU-C1, and SNU-C5 cell lines were purchased from the Korean Cell Line Bank (KCLB, Seoul, Korea); and the DLD-1 and HT-55 cell lines were purchased from American Type Culture Collection (ATCC, Manassas, VA, USA) and Sigma-Aldrich (St. Louis, MO, USA), respectively. DLD-1, HCT 116, HT-29, KM12, SW403, and SW480 cells were maintained in RPMI 1640 medium (HyClone Laboratories, Logan, UT, USA) containing 10% fetal bovine serum (FBS; HyClone Laboratories) and 1% penicillin/streptomycin (PS; Thermo Fisher Scientific, Waltham, MA, USA). HT-55 cells were maintained in MEM (HyClone Laboratories) containing 10% FBS (HyClone Laboratories) and 1% PS (Thermo Fisher Scientific). SNU-C1 and SNU-C5 cells were maintained in RPMI 1640 medium containing 25 mM HEPES (HyClone Laboratories), 10% FBS (HyClone Laboratories), and 1% PS (Thermo Fisher Scientific). All cell lines were maintained under a humidified atmosphere of 5% CO_2_ and 95% air at 37 °C. The culture media were refreshed every 2 to 3 days.

### 4.6. Cell Viability and siRNA Transfection

siRNA transfection was performed using four pooled On-Target Plus siRNAs to target genes (On-Target Plus Smart Pool siRNA; GE Dharmacon). All cell lines were seeded in a black bottom 384-well plate (Corning, Corning, NY, USA). Each cell line was seeded at a different number of cells per well (DLD-1, HCT 116, HT-29; 800, HT-55, KM12, and SW480 cells; 1500; SNU-C5; 2000, SNU-C1, and SW403 cells; 3000). siRNA reverse transfection was performed in two repeats for 72 h. Negative control siRNAs (siNC; GE Dharmacon) and positive control siRNAs were added (siPLK1; GE Dharmacon) to confirm distinguishing sequence-specific silencing from nonspecific effects and knockdown efficiency, respectively. A control experiment using siPLK1 against Polo-Like Kinase 1 (PLK1), a gene important for the cell cycle, showed that the assay was appropriate for identifying essential genes. Reverse transfection was performed with siRNAs (final concentration 10 nM) and Lipofectamine RNAiMAX (0.1 µL per well; Thermo Fisher Scientific) diluted in Opti-MEM I (Thermo Fisher Scientific). After incubation, the cells were stained with CellTiter-Glo for the cell viability assay (Promega, Madison, WI, USA). After siRNA reverse transfection, the cells were stained with CellTiter-Glo for the cell viability assay (Promega, Madison, WI, USA). The cell viability assay was performed according to the manufacturer’s protocol for the cell. All cell viability assays were performed in two replicates.

### 4.7. Statistical Analyses for Bidirectional Analysis

We used Student’s *t*-test to compare two groups by using R.

### 4.8. Kaplan-Meier Survival Analyses

The RFS of colon adenocarcinoma (COAD) patients over five years was analyzed using the Kaplan-Meier method and log-rank test via R. The patients were divided into high expression and low expression groups based on the median gene expression among all patients for each gene. To obtain a *p*-value indicating the significance of this analysis, a log-rank test was performed.

## 5. Conclusions

This study was focused on finding genes of which mRNA expression was predictive to shRNA and/or sgRNA efficacy on diverse colon cancer cell lines. We integrated large-scale, collateral mRNA expression and RNAi data available from about 40 colon cancer cell lines, and calculated bidirectional cross-association scores (predictivity and descriptivity) for each of approximately 18,000 genes between mRNA and sh/sgRNA data. The mRNA expression of 90 and 74 genes from diverse molecular classes showed significant (*p*-value < 0.01) cross-association scores with shRNA and sgRNA efficacies, respectively. While most of hit genes were inconsistent between shRNA and sgRNA cases, we found six common hit genes (*CHAF1B, HNF1B, HTATSF1, IRS2, POLR2B* and *SATB2*) between them. The experimental validation showed that the anticancer efficacy of siHNF1B treatment were significantly correlated with its mRNA expression level among nine colon cancer cell lines. We further extended our cross-association analysis to patient sample data. *KIF26A* and *ZIC2* gene expression, with which shRNA efficacy displayed significant scores, were found to correlate with the survival rate from colon cancer patient data. Our study demonstrated that bidirectional predictivity and descriptivity calculations between collateral datasets served as simple and effective measures for finding genes with associated two experimental data on diverse cancer samples.

## Supplementary Materials

The following are available online at https://www.mdpi.com/2072-6694/12/11/3091/s1, Table S1: Major functional classes of 20 genes with high RNAi efficacy in Figure 1.
