# Supplementary material for: Analysis of Cross-Association between mRNA Expression and RNAi Efficacy for Predictive Target Discovery in Colon Cancers
Source: Cancers (Basel). 2020 Oct 23;12(11):3091. doi: 10.3390/cancers12113091 (PMC7690798; doi:10.3390/cancers12113091)
Supplement: Supplementary file 1 [file cancers-12-03091-s001.pdf]

Supplementary Materials

Analysis of Cross-Association between mRNA Expression and RNAi Efficacy for Predictive Target Discovery in Colon Cancers

Euna Jeong, Yejin Lee, Youngju Kim, Jieun Lee and Sukjoon Yoon

Table S1. Major functional classes of 20 genes with high RNAi efficacy in Figure 1.

Functional Category	Genes	Full Name	Hit
Transcription	*BUB3*	BUB3 mitotic checkpoint protein	sgRNA
	CTNNB1	catenin beta 1	shRNA
	POLR2L	RNA polymerase II subunit L	sgRNA
	RAN	RAN, member RAS oncogene family	sgRNA
	SF3B2	splicing factor 3b subunit 2	shRNA
	SNRNP200	small nuclear ribonucleoprotein U5 subunit 200	sgRNA
	SNRPD1	small nuclear ribonucleoprotein D1 polypeptide	common
Translation	*EEF2*	eukaryotic translation elongation factor 2	sgRNA
	EIF3B	eukaryotic translation initiation factor 3 subunit B	shRNA
	RPL14	ribosomal protein L14	shRNA
	RPL37	ribosomal protein L37	sgRNA
	RPL5	ribosomal protein L5	shRNA
	RPL7	ribosomal protein L7	shRNA
	RPS15A	ribosomal protein S15a	shRNA
	SARS1	seryl-tRNA synthetase 1	sgRNA
Protein metabolism	*HSPE1*	heat shock protein family E (Hsp10) member 1	sgRNA
	PSMB3	proteasome 20S subunit beta 3	sgRNA
	RBX1	ring-box 1	shRNA
	UBA1	ubiquitin like modifier activating enzyme 1	sgRNA
	USP39	ubiquitin specific peptidase 39	shRNA

© 2020 by the authors. Licensee MDPI, Basel, Switzerland. This article is an open access article distributed under the terms and conditions of the Creative Commons Attribution (CC BY) license (http://creativecommons.org/licenses/by/4.0/).
